# Generalized tetanus in a 4-year old boy presenting with dysphagia and trismus: a case report

**DOI:** 10.1186/1757-1626-2-7003

**Published:** 2009-04-29

**Authors:** Petrus Rudolf de Jong, Thea de Heer-Groen, Cornelis Hendrik Schröder, Nicolaas Johannes Georgius Jansen

**Affiliations:** 1 Department of Pediatric Intensive Care, Wilhelmina Children's Hospital, University Medical Center Utrecht, Home mailbox KG.01.319.0, PO Box 85090, 3508 AB Utrecht, The Netherlands; 2 Department of Pediatrics, Lukas Hospital Center, Apeldoorn, The Netherlands

## Abstract

**Introduction:**

The low incidence of tetanus in developed countries has resulted in a decreased vigilance of this disease. This raises concern, as the prodromal stadium of a generalized tetanus infection may lack the characteristic paroxysmal muscle spasms. Tetanus can rapidly progress into life-threatening muscle spasms accompanied by respiratory insufficiency and/or autonomic dysfunction. This emphasizes the need for early diagnosis and treatment.

**Case presentation:**

A 4-year-old Caucasian boy presented with a one-week history of general malaise, mild fever, indolence and anorexia. He subsequently developed dysphagia, sialorrhoea, difficulties opening the mouth and eventually dehydration. Due to parental concerns about the boy's refusal of fluids, a pediatrician was consulted. At that time of presentation he showed signs of trismus and muscle rigidity. Together with the lack of immunization and a toe nail infection, this lead to the suspicion of a generalized tetanus infection. After sedation, endotracheal intubation and ventilation, passive immunization and initiation of antimicrobial treatment, he was immediately transferred to a pediatric intensive care unit (PICU) for further treatment. The frequency and severity of paroxysmal muscle spasms increased progressively during his PICU stay, despite high doses of sedatives. Not before two weeks after admittance, extubation and careful weaning off sedatives was achieved.

**Conclusion:**

Tetanus infection remains a rare but potentially lethal disease in developed countries. As the full scope of classical symptoms may be absent at first presentation, tetanus should always be considered in non-immunized patients with an acute onset of dysphagia and trismus.

## Introduction

Tetanus is a neurotoxin-mediated disease characterized by a progressive spastic paralysis of multiple muscle groups. The neurotoxin (tetanospasmin) disrupts neurotransmitter release in inhibitory neurons, leading to peripheral muscle rigidity and spasms. Tetanospasmin is produced by the obligate anaerobic species *Clostridium tetani*, of which its spores are ubiquitously distributed in our environment. This causes the inevitable increased risk of tetanus infection after wound contamination. Although tetanus has become rare in developed countries due to the successful implementation of primary immunization series, infants that are not immunized on religious or philosophical grounds are still at risk [1].

Muscle rigidity and spasms constitute the typical clinical hallmarks of generalized tetanus *e.g.* trismus (lockjaw) and opisthotonus [2]. Importantly, the onset of a generalized tetanus infection is not always associated with the clinical signs described above. Tetanus presenting with solely oropharyngeal symptoms can be misdiagnosed as a more common oropharyngeal infection (*i.e.* peritonsillar abscess). However, unrecognized tetanus may rapidly progress into a critical condition with severe muscle spasms, autonomic dysfunction and/or respiratory failure [3]. Patients with a clinical suspicion of tetanus must therefore receive local wound care, tetanus immunoglobulins plus antimicrobials and be transferred to a specialized intensive care unit without any delay. We report a child who presented with general malaise, anorexia, dysphagia, trismus and dehydration, which rapidly developed into severe generalized tetanus.

## Case presentation

A 4-year-old Caucasian boy presented to a regional hospital with a one-week history of general malaise, indolence, mild fever and progressive anorexia. Three days prior to presentation at the hospital he had started to refuse all food and fluids, accompanied by a progressive dysphagia, sore throat and sialorrhoea. An otorhinolaryngologist had been consulted two days before presentation, who had considered a peritonsillar abscess. However, his examination at that time did not provide any clues for an oropharyngeal infection. Subsequently, the boy demonstrated increased difficulties with opening his mouth and experienced a progressive dehydration. Due to the parents' concern about the refusal of fluids and dehydration, a pediatrician was consulted.

The history revealed that the boy had recently injured his left hallux. This had resulted in a small local hematoma and loose toenail. There were no recorded insect or animal bites. Based on religious grounds, the boy had not received immunization according to the Dutch National Immunization Program. The other children, including his identical twin, were healthy.

On physical examination in the regional hospital we saw an afebrile, irritable and anxious boy gently playing at the table, with trismus and mild dehydration. After being asked to walk, he showed muscle spasms of the back and thighs evidently worsening during examination. There was no cervical lymphadenopathy and the ear and nose examination was unremarkable. Inspection of the oropharynx was not possible due to trismus. Tendon reflexes were normal, there was no meningeal irritation. The loose toenail did not show clear signs of inflammation. The heart rate was slightly increased, the blood pressure was normal and further clinical examination was unremarkable. The initial differential diagnosis included oropharyngeal infections (*e.g.* tonsillitis, peritonsillar abscess), botulism, rabies, strychnine poisoning, hypocalcemia, psychogenic causes and tetanus. Based on normal complete blood cell count and chemistry profiles, immunization status and the presence of generalized muscle spasms and a possible portal of entry, the working diagnosis 'generalized tetanus' was established.

**Figure 1:**
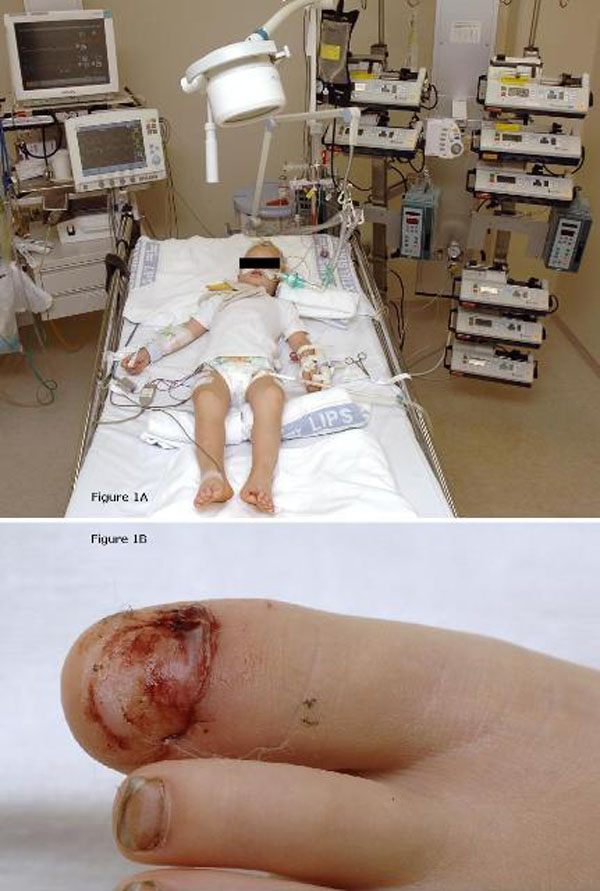
**(A) A 4-year-old caucasian boy with generalized tetanus at the time of admittance to the pediatric intensive care unit where mechanical ventilation, deep sedation and extensive cardiorespiratory monitoring were performed**. **(B)** More detailed photograph of the left hallux toenail, in this case the most likely portal of entry, after surgical debridement.

**Figure 2:**
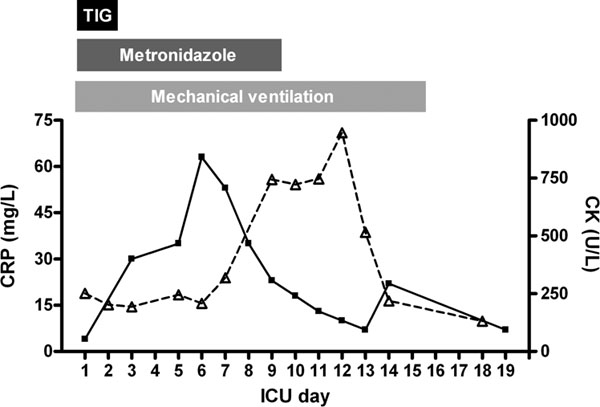
**C-reactive protein (CRP; filled squares) levels reached a maximum at the end of the first week of PICU stay**. In contrast, creatine kinase (CK; open triangles) levels only started to increase in the second week of admittance. Maximal creatine kinase levels were measured at the end of the second week and were accompanied by increases in the severity of muscle spasms and trismus. PICU: Pediatric Intensive Care Unit; TIG: anti-tetanus immunoglobulins.

Treatment was initiated immediately with the administration of anti-tetanus immunoglobulins (3000 IU i.m.) and amoxicillin (100 mg/kg i.v.). In order to prevent respiratory failure, the boy was intubated and mechanical ventilation was started. Thereafter the boy was transferred to the pediatric intensive care unit (PICU) Figure 1A) for further treatment. The antibiotic regimen was then converted to metronidazole (30 mg/kg/day i.v.) for 10 days in accordance with local guidelines. On the second day of admittance, surgical debridement of the left hallux toenail was performed (Figure 1B). In addition, the second intramuscular dosage of anti-tetanus immunoglobulins was administered. Active immunization against diphteria, tetanus and polio (DTP) was started after one week. Despite evident clinical signs of tetanus, repeated blood and wound cultures were negative for *C. tetani*. During the entire stay at the PICU repeated cultures of blood, urine and tracheal aspirates remained negative and white blood cell counts remained unremarkable. In contrast, CRP levels increased to a maximum of 63 mg/L at day 6 of PICU stay (Figure 2).

In the second week, the frequency and severity of muscle spasms and trismus significantly worsened. Dosages of midazolam and morphine (i.v.) were increased and clonidine and lorazepam were added to the regimen. In this period, creatine kinase levels were maximal on day 12 at 945 U/mL [reference value: 15-175 U/mL] (Figure 2). Due to anxiety, haloperidol was started. The frequency of muscle spasms and anxiety decreased in the third week, after which gradual weaning off sedatives was started. Uneventful extubation was performed at day 16. Two short periods with increased muscle spasms occurred thereafter, which were successfully treated with diazepam. On day 21 he was transferred back to the referring hospital where he was discharged after 9 days. During regular follow-up visits to the outpatient clinic, no adverse long-term effects were registered.

## Discussion

In developed countries, national immunization programs have succesfully decimated the number of tetanus infections during the previous four decades [4]. In particular, generalized tetanus infections within the first month after birth (tetanus neonatorum) have become extremely rare in the Western world [1]. However, the widespread distribution of *C. tetani* spores in our environment in combination with the lack of herd immunity still leads to incidental tetanus infections in non-immunized individuals. This case report urges on considering generalized tetanus infection, also in developed countries, when confronted with non-immunized children who present with unexplained oropharyngeal symptoms.

In our patient, dysphagia, sore throat and difficulty opening the mouth (with the suspicion of a peritonsillar abscess) were the main complaints in the prodromal stadium of severe generalized tetanus. These and other complaints *i.e.* neck stiffness are common in general practices and are rarely regarded as early signs of tetanus. Set against this clinical picture in the prodromal stadium, patients in developing countries are more likely to present with progressed and unambiguous symptoms *i.e.* severe spasms of the facial musculature (risus sardonicus) and opisthotonus [2].

In literature, only two adult case reports demonstrate the diagnostic challenge with oropharyngeal symptoms in generalized tetanus in developed countries [5,6]. In both cases, the patients presented with isolated symptoms of dysphagia and trismus, and generalized tetanus infection was not recognized at first. The initial diagnostic confusion was soon followed by rapid clinical deterioration with either respiratory failure [5] or autonomic dysfunction [6], necessitating prolonged intensive care. These reports emphasize the challenge of diagnosing generalized tetanus infection in the mere presence of dysphagia and trismus, which is accompanied by an undiminished risk of rapid clinical deterioration.

Another complicating matter is the distinction with localized tetanus, in which the distribution of tetanus toxin and muscle spasms is limited to specific body areas. Although these cases are usually associated with good outcome, rare cases with involvement of the cranial nerves (cephalic tetanus) have a high risk of progressing to generalized tetanus with a high mortality [2]. In tetanus neonatorum, the early symptoms include suckling and feeding problems, vomiting and seizures. These often rapidly progress into generalized spasms, opisthotonus or even septic complications [1,2].

Appropriate treatment of generalized tetanus consists of neutralization of free circulating tetanus toxin, surgical debridement, eradication of the bacterial load and advanced supportive care. Human tetanus-specific immunoglobulin (TIG) is available for neutralization of tetanospasmin [7], although there are contradictory reports about the optimal dosage and route of administration [8]. A recent meta-analysis showed that a combination of intramuscular and intrathecal TIG administration is superior to intramuscular treatment alone with regard to mortality from tetanus [9]. Pharmacological eradication of *C. tetani* bacilli can be achieved by either penicillin or metronidazole based regimens. A randomized clinical trial that compared these antibiotics for the treatment of generalized tetanus showed no differences with regard to in-hospital mortality or autonomic dysfunction [10]. In fact, the most contributing factor to reduce mortality from generalized tetanus is treatment within modern (pediatric) intensive care units (ICU) with aggressive sedation protocols and advanced ventilatory support. Benzodiazepine derivatives are the mainstay for sedation in the ICU during the course of generalized tetanus [11]. Autonomic dysfunction remains the major clinical challenge, as hypotension, arrhythmia and cardiac arrest are important predictors of fatality [12]. Multidrug regimens are imperative for the optimal management of autonomic dysfunction, although magnesium sulphate may reduce the need for other pharmacological agents [13].

## Conclusion

The diagnosis of generalized tetanus in children remains a diagnostic challenge in developed countries, as the classical symptoms may be absent at presentation. Early recognition and immediate initiation of advanced critical care are necessary to prevent rapid clinical deterioration. Therefore, the differential diagnosis of non-immunized children with an acute onset of dysphagia and trismus should always include generalized tetanus.

## Consent

Written informed consent was obtained from the parents of the patient for publication of this case report and accompanying images. A copy of the written consent is available for review by the Editor-in-Chief of this journal.

## Competing interests

The authors declare that they have no competing interests.

## Authors' contributions

PRJ wrote the manuscript and was involved in the clinical care. TAHG and NJGJ provided clinical care to the patient and revised the manuscript. CHS was involved in the design and preparation of the manuscript. All authors read and approved the final manuscript.
